# Some Essentials of Preventive Medicine1Read at the forty-first annual meeting of the Medical Association of Central New York, held at Buffalo. October 27. 1908. — Edited by William C. Krauss, M. D.

**Published:** 1909-02

**Authors:** I. M. Slingerland

**Affiliations:** Fayetteville, N. Y.


### Some Essentials of Preventive Medicine.[1]

By I. M. SLINGERLAND, M. D., Fayetteville, N. Y.

EVER since the dawn of civilisation in every department of human endeavor there has always been an age of foundation, of transition, and of renovation,—so in the medical world. Until recently nearly the whole object of scientific medicine has been curative or alleviative but its watchword for the future will be preventive, although curative and alleviative will have to be employed for centuries, because its progress will be hindered by lack of unity and organisation in the profession, political corruption and the niggardliness of municipalities and nations to provide adequate means to carry the work to its fullest and richest perfection.

The old saying, "an ounce of prevention is worth a pound of cure," would lead us to reason, that it is worth at least sixteen times as much to prevent disease as to cure it. Hence, our fees should be sixteen times as much as they are now for cure. In fact, we get only a very small fractional part, because physicians have not educated the public to pay for such services, as well and promptly as they have been educated to pay in all other lines. Hence, the business side of medicine should be taught in all medical schools.

Medicine is not what it was,—less of empiricism and more of an exact science,—so Voltaire's definition of medicine does not apply with as much force today. He said, "one balances upon a foothold of which he knows little, to reach toward a spot of which he knows less, and brings up on another of which he knows nothing."

What are some of the essentials to make preventive medicine as nearly perfection as possible? I can only mention a few and those very briefly. One of the most, if not the most important is the begetting of life. None of us have the say of our individual existence, but we all have something to say about other lives. None ought to be allowed to marry or procreate, who in any way are afflicted with an incurable, contagious or communicable disease. Perfect specimens of humanity should be the foundation of the race. We should encourage the raising of strong, vigorous, healthy children, thereby making the race more immune to disease. Each one's opsonic index would be normal and would remain so with proper care. If the present state of society continues to exist or grows worse, opsonines will have to be injected frequently to keep our miserable lives a little longer on earth than they otherwise would stay. The race would

1. Read at the forty-first annual meeting of the Medical Association of Central New York, held at Buffalo. October 27, 1908.—Edited by William C. Krauss, M. D.

finally become as in ancient times, when sin was everywhere present and salvation was nearly swallowed or burned, to arise again from the ashes, as there were a few seeds left that neither sin nor fire could destroy. Such reasoning is not approved by all, as they say it would interfere with a person's individual right to select his own life partner, but by the facts of life as brought out by the papers and other sources, a great army of men and women are not capable of making a proper selection of their helpmate. It seems to me that marriage with that class is considerable sentimentality, a very little love, much lust and ultimately divorce, suicide or murder. If marriage was harder to contract there would be less wrecks, and divorce less easy, each would find his or her proper mate, before taking the final step. Again, no habitual criminal, or drunkard, or mental defective, or one who is able and yet absolutely refuses to work, should be allowed to marry or have children. If married or not, they should be unsexed or made sterile as such parentage breeds similar, which will ruin any nation if let go to the extent and with the pace we are now traveling.

Jonathan Edwards's gift to humanity in his 1,400 noble descendants had only one among the number that in any way disgraced the name and that was Aaron Burr. But this gift is counterbalanced, or more so, by the 1.500 criminals that sprang from one pair of English criminals. The forces of evil are continually striving to pull down and destroy all normal healthy life. The only way to counterbalance the result is by rearing the best possible stock. Diseased parentage brings forth diseased children or ones susceptible thereto.

Physicians are compelled by law to report all contagious and infectious diseases, and they ought to report all communicable diseases. You do not interfere with their personal liberty in the one case more than in the other, though that would be the cry. One's personal liberty ought to end when that liberty diseases and destroys his neighbor's life and those entrusted to his care. The law that prohibits a physician from divulging the secrets of his patients, necessary for their treatment, ought to be amended so far as it relates to venereal diseases.

Which one of us, if we had a daughter interested in or about to marry a man who had sometime or other had a venereal disease, would not warn her of her danger, law or no law. You would require of him an absolute guarantee of his perfect recovery, before any marital relations would meet with your approval. Would not every true parent be willing to pay large sums, commensurate with their means, to have you save their daughter? It is as much murder not to save a pure, innocent girl, as it would be to make her life extinct by a drug, a blow or a bullet, for the diseased state she would most surely get in, if she marries such a one, is nothing but slow murder, besides such diseased state would make her suffer the torments of the damned, and wreck home, family and hopes of eternal life. There was a time when physicians could be held blameless, for they did not know the nature of, nor the destructive power of their insidious work. Now every thing is changed, and knowledge is power to stamp out some of the greatest curses of the race. All married and all of the marriageable age, ignorant of these facts, should be instructed in regard to the dangers and results of diseased unions to themselves and offspring. If they should become diseased after marriage, they should be put in the best possible physical and moral condition at once.

A very important essential would be to have all children taught sex life, as early as they are capable of comprehending, as a fortification, and not have the most important function in the vegetable and animal kingdom, that of reproduction, relegated to the street gammons, and the vile; to teach the boys and girls what should be entrusted only to the most skilled and purest of teachers, physicians and intelligent fathers and mothers. The sex life of the simplest forms of plants should be taught first, and there should be a gradual growth in sex knowledge until its full nature is understood in the highest type of animal life—man. That would be a very great achievement for mankind, as all then would look upon sexuality in its pure sense and would guard it as a priceless blessing, not to be misused or abused, but used as nature intended. Ignorance may be bliss sometimes, but in the matter of sexuality it is disease, death and eternal destruction. On its proper teaching and the proper guiding of the sexes hang the destines of our race.

Perverted sexuality is perverted mentality, morality and spirituality. No good fruit grows without perfect seed. Neither does perfect mankind exist without good healthy parentage. Hence, all schools should have medical inspectors, paid by the state a good salary to look after the physical, moral and mental development of, and the hygiene of all the functions of the child, as well as of the sanitary conditions of the school. When God created man in His image, he was pure and free from disease, but by disobedience, a great part of that image has been lost through disease. Unless these processes are checked by the forces of good, the forces of evil will mar still more that image until man is buried in physical and moral filth.

Another essential is in the matter of charity. Sweet charity in most cases as doled out by municipalities, public institutions, free dispensaries and the like, is nothing but putting a premium on pauperism and crime. Many are led to beg and become paupers and criminals as they can get help so easily. Let a person become dependent once, the chances are he will always be more or less dependent, especially, when an ill wind blows ever so lightly. The really needy and most worthy do not apply until they have exhausted all their resources. It is not charity most want, but sympathy and work. Every one able to work ought to be given it at good living wages, according to the services rendered: Those that won't work ought to starve until willing. I believe in giving all worthy, dependent sick, afflicted and helpless the best possible care so long as really necessary; but when a person uses his birthright in riotous living, becomes sick or injured, can be taken to some public institution, and there receive the best care of physicians and nurses with the best of food, paid for by the hard-working small home-owner, who can not support his own as well, is robbery of the worst kind. When he is turned loose again, he does the same thing knowing he will be cared for as many times as he debauches himself.

All institutions, where pay is taken for care and maintenance, should require and demand something of every one according to his means, no matter how little, and the balance paid by local government, the unworthy and fakirs, just long enough to get them on their feet, then put them to work and make them pay for their whole keep. All able to work, no matter what wages they get, should be compelled to pay a certain per cent. to the government, and their employer compelled to take it out, and pay it to the local government. In that way all would share in the burdens of others. It is a great hardship for the willing and able workers to have to support the great army of paupers, tramps and criminals well able to care for themselves, but who will not work as long as society will support and tolerate them. They are only a menace to society, without one redeeming feature, extracting the life blood of the nation. Just think, 31,000 tramps taken care of in a year in New York State. More nervous wrecks and suicides in the ranks of the willing workers are, not wholly due to their own burdens, but those of the leeches of earth. The old saying, "The world owes me a living, and I am going to have it," is only true so far as you earn it. He that will not work, neither should he eat. "By the sweat of your brow shall ye earn bread," are injunctions that no human power should set aside. I do not believe in the salvation of one, by the damnation of the many.

Prevention of war is a great prevention of disease and death, and hence a very great essential. In war times all the able-bodied men are drawn to be shot down or die of disease, while the diseased, infirm, criminals, cowards, or those who can buy a substitute, are left at home to breed. If a new generation develops hardy, healthy, happy men, the best of the country, a new war must by some pretext be started to kill them off by disease or service. Those no good for any thing or to any one ought to be the first gathered in, forced to the front as a shield and breast-work to protect the genuine army. Then they would go into action with greater love for liberty and country.

Inspection of immigrants, not only at home but abroad, is very essential and should be more rigidly enforced; not alone as to their baggage, moral and physical external condition, but minutely as to their internal conditions. They are capable of sowing the seeds of virulent diseases by their excretions. Let all criminals, paupers, licentious and diseased be excluded forever. We can raise enough of that class.

All domestic animals and their diseases should be carefully studied, and such a thorough knowledge be obtained as will protect the human race from contamination and infection. Our intimacy with them is too close, and the danger too great, not to provide protection.

Sanitation and hygiene carried to the highest proficiency is very essential in preventing disease. Their study should be made obligatory in all schools. Trained specialists should be employed in all capacities that enhance their value as regards the health and life of the race. Every house should have plenty of space, so the sunlight and air could penetrate every room and corner. The distances between them should be according to their height. Dust, dirt and the devil should be carefully excluded. They all defile and death lurks therein. Garbage and other refuse should be made free from noxious germs, or those destructive to man. We must remember that the greater portion of germ life is really life to man. All leguminous plants need bacteria for their perfect and abundant development. Many of the finest products for our comfort and welfare could not exist without them. In fact, life is one grand warfare between constructive and destructive ferments due to bacteria. Pure water should be furnished every individual, no matter where he is. In traveling we are often compelled to take stimulants, as the water is so impure that most believe there is less disease and death in stimulants. The millennium will never come for the temperance people until pure water is every where. Pure food, drink and drugs must be insisted upon if we would prevent many diseases. Physicians must learn to let all such impurities alone, if they would be safe, wise and respected leaders and builders of mankind. Bad examples destroy hope and faith. There are enough pure things in the world, for us all, but we must know what they are and how to keep them pure. If we can only detect and destroy the bad, a great essential is at hand. Purity is the watch word in all departments of life. If it had always been so, many wrecks along the shores of time, would have been a lasting blessing to mankind and would have been endowed with that vigor and purity God gave to man when He created him in His image.

Unity and organisation in the profession is absolutely essential for vigorous and accomplished prevention. Thanks, that the tendency is in that direction. The more united, the better fed, clothed, happy and contented we will become. Little jealousies, animosities, and derogatory sayings are demoralising and mean trouble, and trouble means defeat. Neither do they elevate the profession. The poorest equipped may give the most learned some points of value. If greater unity and organisation existed, no health department would give their services for the small salary they do, no school inspector would labor for two and six pence a day and find himself. Municipalities are very liberal with their law agents, who try to get or keep them out of trouble, but whose services are not worth the millionth part of the health departments. Yet their meager salary is what it is because physicians have not the backbone and united efforts to enforce their just demands. Governments are willing for a principle to pay 4,000 or more dollars for a fifteen cent law suit, but wish to pay only fifteen cents for their health and life protection. Think of the enormous sums physicians are saving the country by preventive medicine, at the same time taking the bread out of their own and families' mouths. The only case in history where the more proficient a science and art becomes, the less the renumeration. Most any department could be dispensed with better than the health or medical department, yet all other departments of governments get the plums, while the medical get the pits and skins of the diseased fruit. The laborer is worthy of his hire, and as such the physicians should be well paid. If we should act toward each worthy brother in

> Seeing only what is good,
> Tasting only what is sweet,
> Leave the chaff, and take the wheat

We could get any thing demanded for saving the world from disease and pestilence. Who can estimate the value of the medical profession in averting such calamities. Who would not be willing, if properly taught, to pay for prevention from a serious malady, the full price demanded for carrying one through it. Thus avoiding the worries, anxieties, and dangers of the disease. We should work together in a spirit of fellowship and brotherly love, side by side, pulling in the same direction, and at the same time and wondrous results could be obtained.

In working out all the details of preventive medicine, so as to bring it to its highest development; as many or more physicians than now will be required, but they will work more along new lines and receive better rewards; as the state will ultimately pay for all services directly related to the general public health, and in time all physicians will be so remunerated according to their ability, skill displayed and results accomplished. The British Medical Association almost unanimously recommended that all sick persons be treated at public expense, as the physicians would be relieved of the petty wories of fee collection, the stress of competitive commercialism, and the "sweating" of the profession by hospitals, "friendly societies" and similar organisations. So many now are treated at voluntary or state aid institutions. We ought to be remunerated in proportion to our ability to keep the race healthy and vigorous. Lawyers are paid large sums to keep corporations and individuals out of trouble. How much more ought the physicians receive for keeping the health of the community perfect.

All ought to be taught to consult their regular physician at stated intervals to learn if the organs and processes of the body are working normally.

An army of well trained, well paid government investigators are needed and essential, to find out the causes and the best methods of combating diseases. For in knowing causes can only sure prevention result. Investigators who will teach us how to accurately, in each case diagnosticate diseases in their invasion or incubation stages. More accurate technic, illumined signs, delicate sounds, skilfully made, delicate and accurate instruments, not yet conceived of, whereby we can detect the normal and diseased life processes with readiness and accuracy; which would be as much or more of a revelation than the *x*-ray, the causes of malaria, yellow fever, and the like.

During the present century remedies and methods will be better understood and when applied at the right time will cure or alleviate all diseased conditions. Nothing is impossible. We do not yet know how all the complex problems of life will be solved but we are assured, so long as the world is fit for human habitation, the race is vigorous, healthy and morally clean and reason is not dethroned, new ways and methods will ever be brought to light to aid us in our noble warfare.

Opportunity is waiting and knocking at all our doors, for willing, able, hard-working investigators and applicators, at good livings and more or less renown. We should keep the facts of this poem on opportunity before us:

Maker of human destines am I;
Fame, love and fortune, on my footstep wait,
Cities and fields I walk;
I penetrate deserts and fields remote,
And passing by hovel and mart and palace, soon or late,

I knock unbidden once at every gate;
If sleeping, wake; if feasting, rise before I turn away
It is the hour of Fate, and they who follow me
Reach every state mortals desire,
And conquer every foe save death.
But those who doubt or hesitate,
Condemned to failure, penury and woe,
Seek me in vain and uselessly implore,
I answer not and I return no more.

If we grasp the opportunities as they present, we can make hygiene, sanitation, right-living, pure food and drink properly prepared and all the other essentials necessary for perfect prevention, a great lever to help invigorate and prolong the life and happiness of mankind. If we knew ourselves and how to care for our bodies, very few would die of disease. If the workings of all its minutia were visible and seeing diseases while knocking at its doors, and having a knowledge of how to completely bar the door, would be grand achievements of a life time and fitting monuments for their discoverers.

These investigators will teach us how so to educate our senses, they will become so acute that the very seeing, hearing and touching along with the delicate, simple and accurate instruments and methods, that will be devised, we will be able to diagnosticate physiological and pathological processes with greater accuracy and much easier than now. We will be able to see the very workings of the individual cells, and distinguish one disease from another in the very beginning of its entrance into the system. With a perfectly developed race, healthy minds in healthy vigorous bodies, we would be able to solve all mysteries. As the new glass recently made at Jena, by using the invisible ultra violet rays, will photograph double the number of stars in the same space as the old glass, and as most of the analine dyes can now be made by electra chemical action, and numerous other things not dreamed of a few years ago have been accomplished, so will the methods, instruments, glasses and devices discover for us, untold and simple processes of life and death.

If physicians accomplish all they expect in preventive medicine, and mankind learn to treat their fellowmen according to the Golden Rule and the practices and precepts of the Nazarine; wars and pestilence would cease, disease would be eradicated, happiness would be contagious and communicable, worry, nervous prostrations, mental wreckage, suicide and murder would be unknown, life would be prolonged and death only come from age and injury. Death would loose its sting and be swallowed up in victory and be unconscious and joyous as birth.

